# The putative methyltransferase LaeA regulates mycelium growth and cellulase production in *Myceliophthora thermophila*

**DOI:** 10.1186/s13068-023-02313-3

**Published:** 2023-04-03

**Authors:** Zhen Zhao, Shuying Gu, Defei Liu, Dandan Liu, Bingchen Chen, Jingen Li, Chaoguang Tian

**Affiliations:** 1 Key Laboratory of Engineering Biology for Low-carbon Manufacturing, Tianjin Institute of Industrial Biotechnology, Chinese Academy of Sciences, Tianjin, 300308 China; 2 National Technology Innovation Center of Synthetic Biology, Tianjin, 300308 China; 3 University of Chinese Academy of Sciences, Beijing, 100049 China

**Keywords:** *Myceliophthora thermophila*, LaeA, Fungal growth, Cellulase, Gene regulation

## Abstract

**Background:**

Filamentous fungi with the ability to use complex carbon sources has been developed as platforms for biochemicals production. *Myceliophthora thermophila* has been developed as the cell factory to produce lignocellulolytic enzymes and plant biomass-based biofuels and biochemicals in biorefinery. However, low fungal growth rate and cellulose utilization efficiency are significant barriers to the satisfactory yield and productivity of target products, which needs our further exploration and improvement.

**Results:**

In this study, we comprehensively explored the roles of the putative methyltransferase LaeA in regulating mycelium growth, sugar consumption, and cellulases expression. Deletion of *laeA* in thermophile fungus *Myceliophthora thermophila* enhanced mycelium growth and glucose consumption significantly. Further exploration of LaeA regulatory network indicated that multiple growth regulatory factors (GRF) Cre-1, Grf-1, Grf-2, and Grf-3, which act as negative repressors of carbon metabolism, were regulated by LaeA in this fungus. We also determined that phosphoenolpyruvate carboxykinase (PCK) is the core node of the metabolic network related to fungal vegetative growth, of which enhancement partially contributed to the elevated sugar consumption and fungal growth of mutant Δ*laeA*. Noteworthily, LaeA participated in regulating the expression of cellulase genes and their transcription regulator. Δ*laeA* exhibited 30.6% and 5.5% increases in the peak values of extracellular protein and endo-glucanase activity, respectively, as compared to the WT strain. Furthermore, the global histone methylation assays indicated that LaeA is associated with modulating H3K9 methylation levels. The normal function of LaeA on regulating fungal physiology is dependent on methyltransferase activity.

**Conclusions:**

The research presented in this study clarified the function and elucidated the regulatory network of LaeA in the regulation of fungal growth and cellulase production, which will significantly deepen our understanding about the regulation mechanism of LaeA in filamentous fungi and provides the new strategy for improvement the fermentation properties of industrial fungal strain by metabolic engineering.

**Supplementary Information:**

The online version contains supplementary material available at 10.1186/s13068-023-02313-3.

## Background

Filamentous fungi possess the ability to secret enzymes that deconstruct complex polysaccharides and have been developed for the industrial production of cellulolytic enzymes and plant biomass-based biofuels and biochemicals [1–5]. The thermophilic filamentous fungus *Myceliophthora thermophila* (synonym: *Sporotrichum thermophile*), with optimal growth temperature at high temperature (45 °C) [6], is used as an ideal cell factory in industrial biotechnology. Genome and experimental data suggested that this thermophilic mold is capable of producing a large amount of lignocellulolytic enzymes, including the complete set of thermostable carbohydrate-active enzymes (CAZymes) involved in biomass degradation [7–9]. Moreover, the genome-editing technique has been developed to facilitate the in-depth understanding and design of *M. thermophila* metabolic pathways [10–12]. *M. thermophila* has been engineered into a platform to produce enzymes [1, 13], commodity chemicals [2–4], and biofuels [5]. However, low efficiencies of fungal growth and substrate utilization are significant barriers to the satisfactory yield and productivity of target products during fungal fermentation. To improve the fermentation properties of *M. thermophila* as cell factory, it is necessary to elevate mycelium accumulation and cellulose degradation.

The global regulator LaeA was first discovered in *Aspergillus nidulans* in 2004 [14]. A large number of studies have shown that LaeA participates in regulating fungal physiological characteristics, including fungal growth, asexual and sexual development, cellulase production, and secondary metabolism [15–18]. Under light conditions, LaeA suppresses the expression levels of VelB and VeA proteins and promotes the formation of asexual spores. On the contrary, in the absence of LaeA, Velvet protein is not inhibited and sexual development is favored [17]. The loss of *lae1* significantly reduced sporulation in *Trichoderma reesei* and *Trichoderma atroviride*, while overexpression of *lae1* led to increased sporulation [16, 19]. While in *Monascus ruber*, conidia production of Δ*laeA* strains was about thrice that of wild type. The strain Δ*laeA* formed abnormal colonies that had abundant aerial hyphae as compared to *M. ruber* wild type [18]. Moreover, *laeA* disruption channeled more carbon flux towards biomass production in *Aspergillus charcoal* in citric acid fermentation process, resulted in an 80% increase in biomass accumulation in spite of the similar sugar consumption of Δ*laeA* and wild-type strain [15]. In cellulolytic fungi *P. oxalicum* and *T. reesei*, LaeA/Lae1 was essential for cellulase genes expression and its disruption impaired production of cellulolytic enzymes and deprived the fungi of the capability of growing on plant cellulose [16, 20, 21]. It was reported that Lae1, the LaeA ortholog, modulates cellulase gene expression in *T. reesei*, a process dependent on the function of the general cellulase regulator XYR1 [16, 20]. Similarly, in *P. oxalicum*, LaeA is necessary for the expression of cellulase activated by ClrB (an ortholog of Clr-2) [21, 22]. Although LaeA played so important role in fungal growth and cellulase production, the mechanism of LaeA remains largely unclear.

LaeA is a conserved protein widely distributed in fungi (Additional file 2: Fig. S1), possessing a conserved S-adenosylmethionine binding domain common to seven-β-strand methyltransferases, predicted as a putative methyltransferase and might linked to the remolding of chromatin structure to regulate gene transcription by protein lysine or protein arginine methylation of histone [16, 17, 23–25]. In *Aspergillus kawachii*, LaeA controls the expression of CexA (citrate export protein) by adjusting the euchromatin/heterochromatin ratio of the *cexA* promoter region, thereby affecting the production of citric acid [26]. In addition, in cellulolytic fungus, its ortholog Lae1 regulates the expression of polysaccharide hydrolytic enzymes by modifying the chromatin structure of promoter regions of cellulolytic genes [22]. Similarly, *A. nidulans* LaeA was considered to remodel histone methylation levels to regulate expression of secondary metabolism genes [14]. In recent studies, site-specific mutation of S-adenosyl methionine (AdoMet) binding site suggested methyltransferase activity is crucial for normal function of LaeA [27–29]. Consistently, it was found LaeA can be self-methylated at a methionine residue (M207), although this methylation was not crucial for its function [30]. Nevertheless, no substrate for LaeA has been identified and how LaeA regulating fugal physiology by methyltransferase activity is still unclear.

In this study, the regulatory network and regulatory mechanism of the putative methyltransferase LaeA were comprehensively investigated in *M. thermophila*. We demonstrated that LaeA functions as a global regulator, involving in mycelium growth, sugar consumption, cellulases expression, and secondary metabolite. The growth regulatory factors (GRF) Cre-1, Grf-1, Grf-2, and Grf-3, servicing as the repressors of carbon metabolism, were regulated by LaeA. We also determined that enhancement of phosphoenolpyruvate carboxykinase partially contributed to the elevations of sugar consumption and fungal growth in deletion of *laeA* mutant. Furthermore, the global histone methylation assays indicated that LaeA is associated with modulating H3K9 methylation levels. The normal function of LaeA on regulating fungal physiology is dependent on methyltransferase domain.

## Results

### Deletion of *laeA* accelerates fungal mycelium growth and sugar catabolism.

**Fig. 1:**
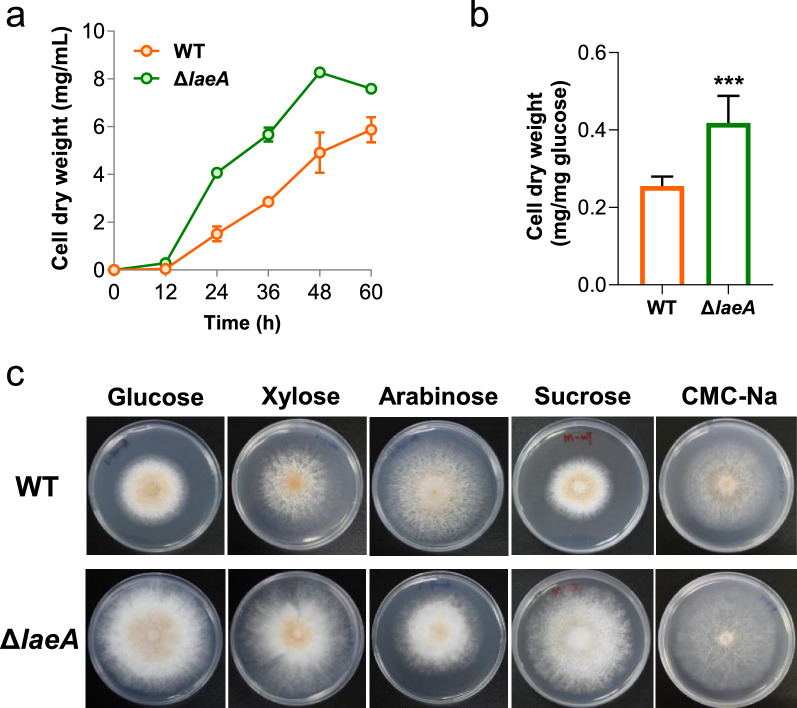
Growth properties of Δ*laeA* strain. **a** Cell dry weight when *M. thermophila* strains grown on 2% glucose. **b** The yield of fungal cell dry weight of strains WT and Δ*laeA* after 36 h of the culture on glucose. **c** Growth properties of *M. thermophila* strains WT and Δ*laeA* on agar plates with different carbon sources, including 2% glucose, 2% xylose, 2% arabinose, 2% sucrose, and 1% sodium carboxymethyl cellulose (CMC-Na). *** represents *p* value < 0.001

In the present study, Mycth_2294559 (LaeA) in *M. thermophila* exhibited a high identity with those in *Trichoderma* and *Neurospora* (Additional file 2: Fig. S1), and its protein sequence showed the presence of four appropriately spaced sequence motifs (I, post-I, II, and III) and S-adenosylmethionine (AdoMet)-binding domain of seven-β-strand methyltransferase [31]. To dissect the function of LaeA, we generated a *laeA* null strain (Δ*laeA*) in *M. thermophila*. When grown on glucose, strain Δ*laeA* exhibited dramatically accelerated the accumulation of cell dry weight and glucose consumption (Fig. 1a, b; Additional file 2: Fig. S2). After 48 h of the growth, the mycelium of Δ*laeA* was 40.6% greater than that of the wild-type (WT) strain (Fig. 1a). The germination rate of Δ*laeA* conidia was also higher than that of the WT. Germlings of Δ*laeA* were readily observable after 4 h, which was approximately 2 h earlier than of the WT strain (Additional file 2: Fig. S3). Next, we continued to check the growth phenotype of Δ*laeA* with other carbon sources, which can also be produced by cellulose degradation, including sucrose, xylose, arabinose, and CMC-Na. Notably, the elevated growth rate was maintained under all tested conditions (Fig. 1c).

### LaeA affects the regulatory network of mycelium growth by regulating the expressions of multiple transcriptional factors

**Fig. 2:**
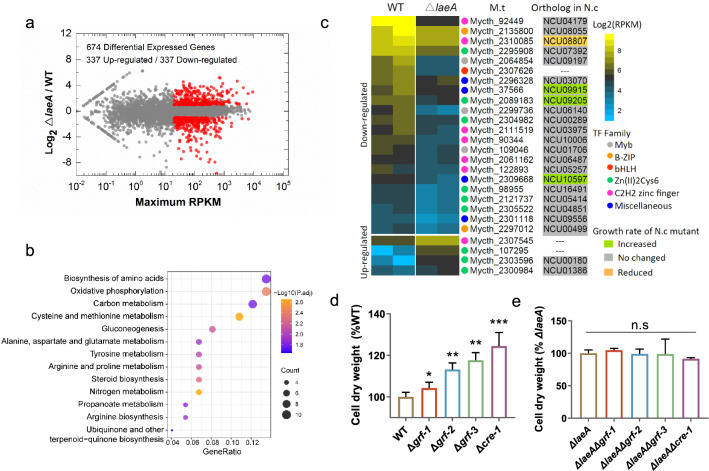
Comparative transcriptomic analysis of strains Δ*laeA* and WT grown on glucose. **a** Differential expression analysis of ∆*laeA* in comparison to WT when growth on 2% glucose for 24 h. Log2 ratio of *∆laeA*/WT vs. maximum RPKM in either strain. Genes exhibiting differential expression levels are plotted in red. **b** KEGG analysis of the 337 genes with up-regulated expression levels in strain *∆laeA* on glucose. **c** Hierarchical clustering of expression profiles of 26 transcription factor genes. Growth phenotype of mutants in *N. crassa* are from ref. [35]. **d** Cell dry weight of *M. thermophila* strains Δ*cre-1*, Δ*grf-1*, Δ*grf-2*, and Δ*grf-3*, after growth on glucose for 36 h. **e** Cell dry weight of double mutants ∆*laeA*Δ*cre1*, ∆*laeA*Δ*grf-1*, ∆*laeA*Δ*grf-2*, and ∆*laeA*Δ*grf-3* after growth on glucose for 36 h. Detailed data for **a**, **b**, and **c** are shown in Additional file 1: Tables S3, S4, and S6, respectively. * depicts *p* value < 0.05; ** depicts *p* value < 0.01 and *** depicts *p* value < 0.001

To explore the mechanism by which LaeA regulates carbon catabolism and mycelium growth, we performed a transcriptional profile analysis of WT and Δ*laeA* strains grown on glucose. When compared with the WT strain, transcription levels of 674 genes were significantly altered in strain Δ*laeA*, with 337 genes having significantly reduced transcription and 337 genes exhibiting significant up-regulation (Fig. 2a; Additional file 1: Table S3). A KEGG analysis of up-regulated genes revealed that these genes were enriched in the functional categories of gluconeogenesis, oxidative phosphorylation, and metabolism and biosynthesis of amino acids (Fig. 2b; Additional file 1: Table S4), consistent with the growth phenotypes of Δ*laeA*. The gluconeogenesis pathway provides intermediates for the synthesis of structural polymers, and its two key enzymes, phosphoenolpyruvate carboxykinase (Mycth_2315623, PCK) and fructose-1,6-bisphosphatase (Mycth_2306943, FBP1), were respectively increased by 12.3- and 2.4-fold in strain Δ*laeA*. As expected, genes encoding glucose transporters (Mycth_98222 and Mycth_112491) were markedly up-regulated in Δ*laeA*. In microbes, efficient sugar transporter is prerequisite for fast carbon uptake and assimilation [32]. These results partly explain the physiological phenotypes of enhanced glucose consumption rate and biomass accumulation of ∆*laeA*. Intriguingly, even though glucose utilization is more efficient in ∆*laeA*, no gene in the pentose phosphate pathway or TCA cycle was significantly up-regulated in the mutant strain and only one gene (Mycth_2316240, encoding phosphoglycerate kinase) in the glycolytic pathway exhibited increased transcription in strain Δ*laeA* (Additional file 1: Table S5), suggesting that central carbon metabolism in *M. thermophila* is mainly regulated at the post-transcriptional level, similar to *S. cerevisiae* and even humans [33].

Transcription factors play an important regulatory role in fungal intracellular metabolism, growth, and development [34]. We thus predicted that transcription factors have major functions in the regulatory network of LaeA. The transcriptional profiles of transcription factors in strain ∆*laeA* were therefore analyzed. Among the 222 genes encoding transcription factors in the genome of *M. thermophila*, 22 were more highly induced in strain WT while 4 exhibited increased expression in strain ∆*laeA* (Fig. 2c; Additional file 1: Table S6). In a previous study, disruption of *N. crassa* orthologs of Mycth_37566, Mycth_2089183, or Mycth_2309668, which had significantly reduced expression levels in strain Δ*laeA*, resulted in elevated linear growth of hyphae on agar plates [35]. In addition, Cre-1 is a major regulator of carbon catabolite repression and regulates the expression levels of genes involved in sugar transport and metabolic activities [36, 37]. In *N. crassa*, deletion of *cre-1* leads to denser hyphae but a lower linear growth rate on agar plates. Similarly, the deletion of *Penicillium funiculosum mig1*, an ortholog of *cre-1*, causes an increase in specific growth rate and glucose consumption [38]. We also found that the expression level of *cre-1* (Mycth_2310085) was decreased by 2.2-fold (*p* value < 0.001) when compared with the parental strain (Additional file 1: Table S3). We; therefore, wondered whether the transcription factors Mycth_37566, Mycth_2089183, Mycth_2309668, and *cre-1* help control mycelium growth connected with the LaeA regulatory network. To test this hypothesis, we constructed single gene mutants of transcriptional factors and analyzed their physiological characteristics. As shown in Fig. 2d, knock-out of the four transcription factors resulted in an increased mycelium growth rate. Therefore, we named Mycth_37566, Mycth_2089183, and Mycth_2309668 as Growth Regulatory Factors (GRFs) Grf-1, Grf-2, and Grf-3, respectively.

To further assess the role of LaeA in regulation of GRF expression, we constructed double mutants of *laeA* and GRF-encoding genes in *M. thermophila*. We observed that the four double deletion mutants phenocopied the strain ∆*laeA* in regard to mycelium growth (Fig. 2e). This result suggests that LaeA and GRFs are connected to the regulatory network of sugar consumption and mycelium growth in fungus.

### Enhancement of gluconeogenesis pathway partially contributed to the elevated sugar consumption and fungal growth of Δ*laeA*

**Fig. 3:**
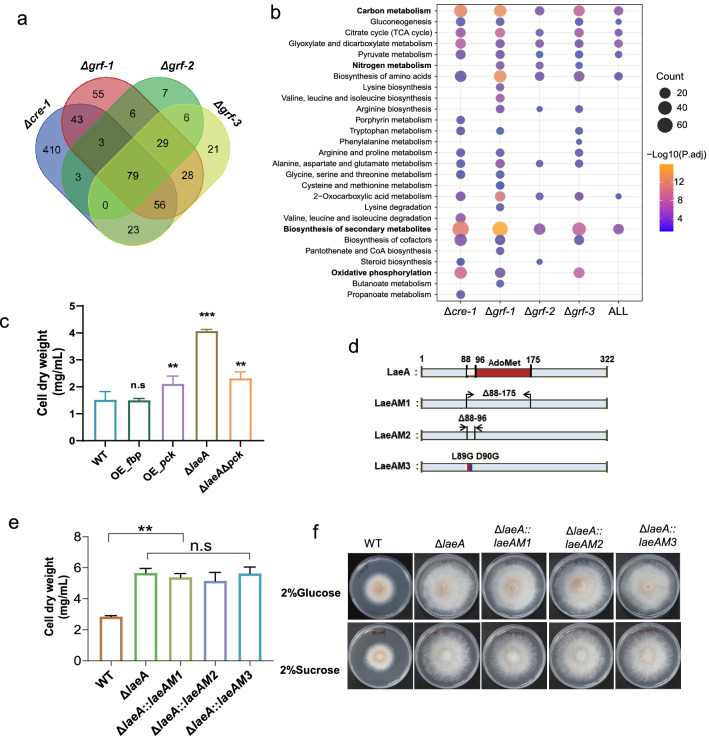
Transcriptomic analysis of the strain with the deletion of GRF genes. **a** Venn diagram showing significantly up-regulated genes in strains Δ*cre-1*, Δ*grf-1*, Δ*grf-2*, and Δ*grf-3*, compared to the WT strain when growth on glucose for 24 h. **b** KEGG analysis of the genes with up-regulated expression levels in strains Δ*cre-1*, Δ*grf-1*, Δ*grf-2*, and Δ*grf-3*. **c** Cell dry weight of strains WT, OE_*fbp*, OE_*pck*, Δ*laeA*, and Δ*laeA*Δ*pck* after 24 h of the cultivation on glucose. **d** Construction of LaeA mutants. **e** Cell dry weight when *M. thermophila* strains grown on 2% glucose for 36 h. **f** Growth properties of *M. thermophila* strains WT, Δ*laeA::LaeAM1*, Δ*laeA::LaeAM2*, and Δ*laeA::LaeAM3* on agar plates for 5 d with glucose, or sucrose as carbon sources. Detailed data for **a**, and **b** are presented in Additional file 1: Tables S7, S8. ** represents *p* value < 0.01; *** depicts *p* value < 0.001

To identify the set of core genes involved in glucose consumption and mycelium growth, we combined transcriptomic analyses of strains Δ*cre-1*, Δ*grf-1*, Δ*grf-2*, and Δ*grf-3*. According to a Venn diagram analysis, 79 genes exhibited significantly increased expression in all mutants, compared with the WT (Fig. 3a; Additional file 1: Table S7). The KEGG analysis indicated that these up-regulated genes were mainly associated with the functional categories of carbon metabolism and nitrogen metabolism (Fig. 3b; Additional file 1: Table S8). Notably, the genes encoding PCK and FBP1 were dramatically induced in all GRF mutants, similar to strain Δ*laeA*; thus, suggesting that PCK and FBP1 are the core nodes of the metabolism network related to fungal vegetative growth.

In view of the fact that high gluconeogenesis pathway activity is required for efficient biomass assimilation, we speculated that the up-regulated expression of *pck* and *fbp* genes was partly responsible for the higher growth rate of Δ*laeA* and the four GRF mutants. To test this hypothesis, we separately overexpressed endogenous *pck* and *fbp* in the WT strain. As shown in Fig. 3c and Additional file 2: Fig. S4, growth phenotypes of the *fbp* overexpression strain resembled that of the WT, whereas enhancement of *pck* expression resulted in more efficient glucose consumption and faster biomass growth. To further assess the role of *pck* in fungal growth, the double deletion mutant Δ*laeA*Δ*pck* were generated, following by testing the growth phenotypes on glucose. Δ*laeA*Δ*pck* showed an obvious decrease in cell dry weight accumulation, as compared to Δ*laeA*, which indicated that PCK has a critical role in the regulatory network of LaeA (Fig. 3c). However, Δ*laeA*Δ*pck* still showed faster growth rate than WT. Taken together, these results reveal that the growth rate and glucose consumption of strain Δ*laeA* and the four GRF mutants was due, at least partially, to elevated gluconeogenesis activity.

### LaeA is involved in modulating the methylation levels of H3K9

LaeA possesses a conserved S-adenosylmethionine binding domain common to seven-β-strand methyltransferases and is predicted as a putative methyltransferase [16, 17, 23–25]. To identify whether methyltransferase activity is essential in LaeA regulation function, we constructed three mutants of the LaeA protein: LaeAM1 with a deletion of the whole SAM-binding domain (88th–175th amino acid residues), LaeAM2 with a deletion of the conserved S-adenosyl methionine (AdoMet) binding site (motif I, whose disruption completely abolished the HMTase activity [39]) (88th–96th amino acid residues), and LaeAM3 with two points mutation (L89G and D90G) (Fig. 3d). After insertion of *laeAM1*, *LaeAM2*, or *laeAM3* into the *laeA* locus of strain Δ*laeA*, growth phenotypes of Δ*laeA::LaeAM1*, Δ*laeA::LaeAM2*, and Δ*laeA::LaeAM3* strains consistently resembled that of the parental strain Δ*laeA* (Fig. 3e, f). According to these data, methyltransferase activity is essential for LaeA in the regulation of mycelium growth, even though the methyltransferase target is unknown.

**Fig. 4:**
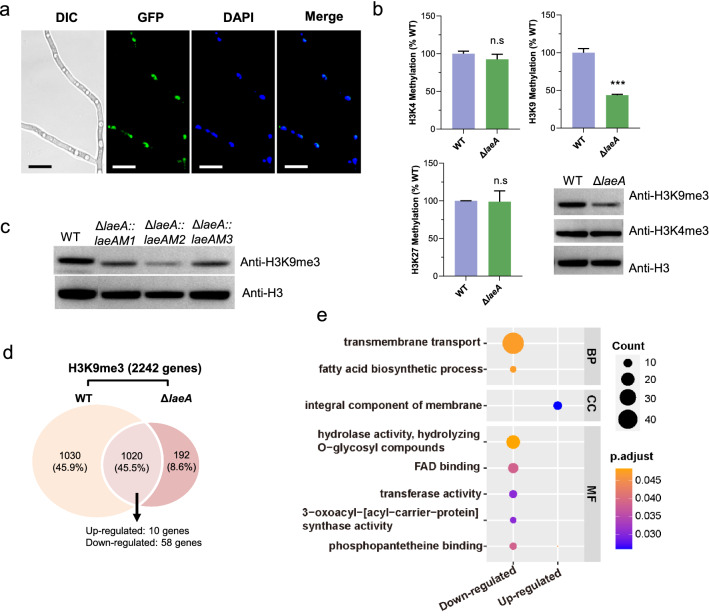
LaeA is involved in modulating the methylation levels of H3K9. **a** *M. thermophila* LaeA protein localized to the nucleus. GFP was fused to the *C*-terminal end of LaeA. Nuclei were stained with the DNA-specific dye 4, 6-diamidino-2-phenylindole (DAPI). **b** Global methylation levels of H3K4, H3K9, and H3K27 in histone protein extracts from strain WT and Δ*laeA* when grown on glucose for 24 h. Western blot assay of extracted histone of WT and Δ*laeA* strains, which were carried out with specific antibodies for histone H3, H3K4me3, and H3K9me3. **c** Western blot assay of extracted histone protein from strains WT, Δ*laeA::LaeAM1*, Δ*laeA::LaeAM2*, and Δ*laeA::LaeAM3*, with a specific antibody for H3K9me3. **d** Chromatin immunoprecipitation followed by sequencing with antibodies against H3K9me3 of strains Δ*laeA* and WT, after growth on glucose for 24 h. Venn diagram exhibiting amounts of genes which regions showing H3K9 trimethylation peaks (Total 2242 genes; 2050 genes in WT strain and 1212 genes in Δ*laeA*,). **e** GO analysis of genes with differential H3K9me3 levels in Δ*laeA* relative to WT as determined by chromatin immunoprecipitation sequencing (ChIP-seq). Down-regulated, representing genes with down-regulated trimethylation level in Δ*laeA*; Up-regulated, representing genes with up-regulated trimethylation level in Δ*laeA*

Previous studies have suggested that LaeA and its orthologs have been postulated to function by modulating methylation levels of histone H3 lysine 4 (H3K4) or lysine 9 (H3K9) [26, 40]. According to our results, LaeA regulation function is dependent on methyltransferase activity and LaeA was translocated into the nucleus of *M. thermophila* (Fig. 4a). We speculated LaeA can modulate methylation levels of histone to regulate fungal properties. To test this hypothesis, we assayed global methylation levels of H3K4, H3K9, and H3K27 in histone extracts from strains WT and Δ*laeA*. As shown in Fig. 4b, total methylation levels of H3K4 and H3K27 in strain Δ*laeA* were comparable to those in strain WT, whereas overall levels of H3K9 methylation were significantly reduced, consistent with the western blot analysis (Fig. 4b). Furthermore, H3K9me3 levels of the mutants Δ*laeA::LaeAM1*, Δ*laeA::LaeAM2*, and Δ*laeA::LaeAM3* were still lower than that in the WT during western blot analysis (Fig. 4c). According to these results, LaeA is involved in the regulation of H3K9 methylation directly or indirectly.

To explore the effect of the changes of the H3K9 methylation, we carried out chromatin immunoprecipitation followed by sequencing (ChIP-seq) in strains WT and ∆*laeA* when grown on glucose. As shown in Fig. 4d, 2242 genes (24.6% of the total) exhibited H3K9 tri-methylation in at least one of the two strains (Additional file 1: Table S9). In the ∆*laeA* strain, 1,088 genes with a decreased H3K9me3 level were enriched in the GO functional categories of transmembrane transport and fatty acid biosynthetic process (Fig. 4e). Taken together, these results demonstrated that methylation modification of H3K9 can affect gene expression by changing chromatin structure in *M. thermophila.* However, H3K9me3 modification at the promoter regions of GRF genes (*grf-1*, *grf-2*, *grf-3*, and *cre-1*) and *pck* were not significantly altered in strain ∆*laeA*, although transcription levels of these genes were significantly changed. These results implied that the expression of four GRF genes and *pck* was indirectly regulated by the methylation modification of H3K9.

### *M. thermophila* LaeA dynamically regulates expression of cellulase on cellulose, dependent on methyltransferase activity

**Fig. 5:**
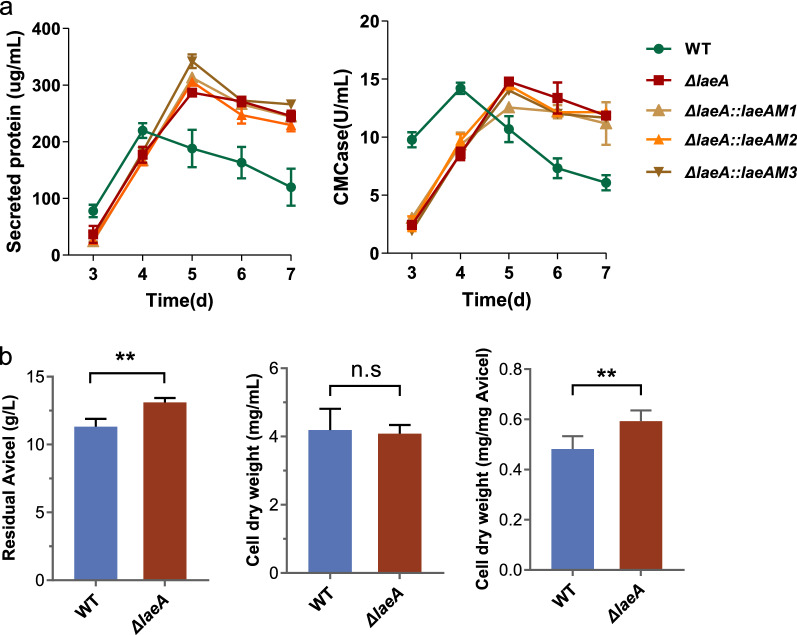
Phenotypic analysis of the strain ∆*laeA* on cellulose. **a** Total extracellular protein concentration, and endo-glucanase activity of *M. thermophila* strains on 2% Avicel. **b** Residual Avicel, cell dry weight, and cell dry weight related to consuming Avicel were measured after 2 d of the cultivation on Avicel. ** represents *p* value < 0.01

In cellulolytic fungi *P. oxalicum* and *T. reesei*, LaeA/Lae1 was essential for cellulase genes expression and its disruption impaired production of cellulolytic enzymes and deprived the fungi of the capability of growing on plant cellulose [16, 20, 21]. To investigate the effects of LaeA on cellulose degradation in *M. thermophila*, we detected secreted protein and hydrolytic activities in the cultures of strains WT and Δ*laeA* with Avicel the sole carbon source. As shown in Fig. 5a, after 3 days of the growth on Avicel, Δ*laeA* exhibited a significant decrease in secreted proteins (~ 30%). As expected, deletion of *laeA* caused a 68% decrease in the CMCase activity when compared with that of the WT. Although consumption of cellulose (Avicel) was reduced by 20.6% in the culture of Δ*laeA* responding to Avicel, Δ*laeA* accumulated a similar amount of biomass as that of the strain WT (Fig. 5b), which further supported that LaeA was involved in regulation of mycelium growth.

However, unlike the previous reports in *Trichoderma* and *Pencillium* [16, 21], secreted protein and endo-glucanase activity in the supernatant of the strain WT culture continued to increase during the first four days, but rapidly declined thereafter. Although Δ*laeA* also constantly produced secreted protein and endo-glucanase with prolonged culture time and peaked at 5th day. The peak values of extracellular protein and endo-glucanase activity in the culture of Δ*laeA* were 30.6% and 5.5% higher than that of strain WT, respectively. Additionally, secreted protein and endo-glucanase activity in the supernatant of the strain Δ*laeA::LaeAM1*, Δ*laeA::LaeAM2*, and Δ*laeA::LaeAM3* strains consistently resembled that of the parental strain Δ*laeA* (Fig. 5a), indicating that LaeA regulation function on cellulase production is dependent on methyltransferase activity.

### Transcriptomic profiles and exoproteomic analysis of Δ*laeA* mutant response to cellulose

**Fig. 6:**
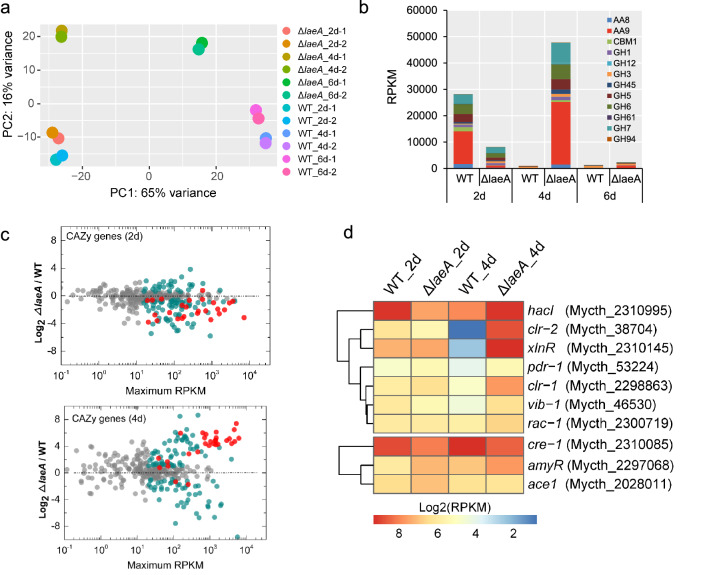
Transcriptomic profile of Δ*laeA* strain grown on Avicel. **a** Principal component analysis of RNA-Seq data from the WT, and Δ*laeA* strains grown on 2% Avicel for 2, 4, and 6 days. **b** Total RPKM value of CAZyme genes from RNA-seq data in WT and Δ*laeA* strains. **c** Relative mRNA abundance of all CAZyme genes in Δ*laeA* mutant vs WT when growth on cellulose for 2 or 4 d. Genes showing significant differential expression are shown in turquoise (based on the adjusted *p* value). Cellulase genes showing differential expression are shown in red. **d** Heatmap analysis of expression levels of transcription factors regulating cellulase production in strains WT and Δ*laeA*. Positive regulator (top); negative regulator (bottom)

To further investigate the dynamically regulation of LaeA on cellulase production of *M. thermophila*, we performed transcriptional profiling of strain WT and Δ*laeA* cultures grown on cellulose for different time periods (2 d, 4 d, and 6 d). A principal component analysis (PCA) indicated the biological replicate the samples from the same time point were clustered tightly, confirming that the RNA-seq data were highly reproducible (Fig. 6a). The analysis of the transcriptional profiles revealed that transcript levels of the genes encoding CAZymes such as GH3, GH5, GH6, GH7, AA8 and some members of the AA9 family were significantly lower at 2 d, but dramatically up-regulated in Δ*laeA* at 4 d, compared with these of the WT strain (Fig. 6b), which was consistent with dynamic changes of protein and cellulolytic enzyme activities in the cultures of strain Δ*laeA* grown on cellulose (Fig. 6a–c). After 6 d of the cultivation, carbon source (Avicel) had been completely consumed, which led to the reduction of cellulase production.

When grown on Avicel for 2 days, transcriptional levels of 467 genes were significantly up-regulated and 491 genes indicated dramatically decreased expression levels in strain Δ*laeA*, as compared to the strain WT. Of these down-regulated genes, 64 genes encoded CAZymes in the glycoside hydrolase family (GHs), glycosyltransferases family (GTs), polysaccharide lyases family (PLs), cellobiose dehydrogenases (AA8, CDH), and lytic polysaccharide monooxygenases (AA9, LPMO) (Fig. 6c; Additional file 1: Tables S10, S12). In addition to cellulose degradation genes, the genes associated with protein synthesis and secretion were also significantly down-regulated in Δ*laeA*, including three encoding the translocation proteins (Mycth_2312007, Mycth_2316209, Mycth_2309221), two encoding ER chaperones (Mycth_2300892 and Mycth_80427) and two essential transcription factors (Mycth_90344 orthologous to *N. crassa* NCU10006 and Mycth_2310995 orthologous to *N. crassa* HacI). In *N. crassa*, Hac-1 is involved in the Unfolded Protein Response (UPR) and essential for the growth on cellulose [41, 42]. As expected, *pck* exhibited an approximately tenfold expression increase in strain Δ*laeA*, thus further verifying the core role of PCK in the regulatory network of growth properties. Also 40 genes involved in oxidative phosphorylation and 27 genes related to ribosome synthesis were highly induced, which explained the similar cell dry weight of Δ*laeA* as the WT strain, despite of lower cellulose degradability. In addition, one gene encoding the essential transcription factor AmyR (Mycth_2301920), ortholog of *N. crassa* COL-26 negatively regulating cellulase secretion [43], was higher induced in strain Δ*laeA*.

In contrast to RNA-seq data at day 2, *∆laeA* showed increased expression of genes encoding CAZymes, including 46 GHs, 11 GT, 7 CBMs, two cellobiose dehydrogenases (AA8), and 11 LMPOs (AA9) as compared to WT, when cultured on cellulose for 4 d (Fig. 6c, Additional file 1: Tables S11, S12), which was not observed in *T. reesei* and *P. oxalicum* [16, 21, 22]. A Funcat analysis indicated that the set of 920 up-regulated genes was significantly enriched in the functional categories of C-compound and carbohydrate metabolism, fermentation, cellular import and two-component signal transduction system (sensor kinase component). The gene (Mycth_114107, with a RPKM > 5000) encoding cellodextrin transporter, ortholog of *N. crassa* Cdt-2 [44], showed 283-fold increase in strain *∆laeA*. The expression of five genes encoding the permease involved in uptake of amino acid (Mycth_2308145, Mycth_2055845, Mycth_2316511, Mycth_2093040, and Mycth_2299723), were more highly induced in strain *∆laeA*. In addition, the genes related to protein secretion were highly induced in mutant of *laeA*, such as Sec2 (Mycth_2310888), Sec5 (Mycth_2298813), Sec53 (Mycth_2294943), and Sec72 (Mycth_2314749), which is an indicative of a need to adapt the secretory machinery to increase production of hydrolases when grown on cellulose. The additional presence of factors involved in ER-associated stress response (RESS) in strain *∆laeA* such as transcription factor Hac1 and endoribonuclease IRE-1 (Orthologous to *N.crassa* NCU02202 [45]), is a further indication (Additional file 1: Tables S11, S12).

The production of cellulolytic enzymes is dependent on transcription factors in fungi and a broader search for transcription factors involved in cellulose degradation has been discovered, such as transcription activators Clr-1/2, XlnR, Hac1, Prd-1, Vib-1, and Rac-1, and transcription repressor Cre-1, AmyR, and Ace1 [46–48]. When dynamic changes in the expressions of cellulase during batch culturing of Δ*laeA* were determined, we found that expression of cellulase activators, Clr-1, Vib-1, and HacI, displayed significantly reduced expression levels (Adjusted p value < 0.05) in Δ*laeA*, while the cellulase repressors, AmyR and Ace1, showed increased expression levels when grown on Avicel for 2 d, which was consistent of the lower expression of CAZyme genes. In addition, Clr-1/2 (Mycth_2298863 and Mycth_38704) and XlnR (Mycth_2310145) in Δ*laeA*, were comparable with that in strain WT on 2nd day, but were dramatically up-regulated at 4th day, which was consistent with the pattern of cellulase synthesis (Fig. 6d). However, in strain Δ*laeA*, *cre-1* exhibited lower expression levels at 2nd and 4th days, which suggested that LaeA could regulated the expression of *cre-1*. To further reveal the dynamic changes in the production of cellulase by strain ∆*laeA*, we assayed transcriptomic landscapes of all 28 putative methyltransferase genes. One gene (Mycth_2295350, named as *laeB*) encoding LaeA-like methyltransferase showed dramatically increased expression levels in strain ∆*laeA* after the culture of 2 d and 4d (Additional file 2: Fig. S5a). We hypothesize that this phenomenon in *M. thermophila* might result from complementary of *laeA* deletion by *laeB* at the last stage of cultivation on cellulose. But, the mutant with the deletion of *laeA* and *laeB* displayed comparable secreted protein and endo-glucanase with strain *∆laeA* (Additional file 2: Fig. S5b).

### LaeA regulates secondary metabolism in *M. thermophila*

LaeA has been identified as a global regulator of secondary metabolism, and mutation of *laeA* impairs the synthesis of secondary metabolites, including pigments [14, 21, 49–51]. In the present study, the culture of the WT strain grown on Avicel gradually turned brown, whereas strain Δ*laeA* remained whitish gray until the end of fermentation (Additional file 2: Fig. S6a). In contrast, both strain cultures were whitish gray under glucose conditions. These results indicate that pigment production is dependent on growth environment and that LaeA is involved in the regulation of secondary metabolism in *M. thermophila*.

During fungal synthesis of secondary metabolites, polyketide synthases (PKSs), nonribosomal peptide synthetases (NRPSs), and terpene synthases (TSs) were responsible for catalyzing synthesis of the core structure. Consistent with the reduced pigment production observed above, transcriptomic analysis revealed that expression levels of three PKS genes and two NRPS genes, necessary for melanin synthesis, were reduced as a result of the deletion of *laeA* (Additional file 2: Fig. S6b and Additional file 1: Table S13). The positive regulation of PKS and NRPS genes by LaeA implied by this result is consistent with observations in *T. reesei*, *Aspergillus*, and *Fusarium* [20, 51–53]. In addition, five genes encoding P450 and three genes encoding short chain dehydrogenase/reductase, which help catalyze modification of the core structure of fungal secondary metabolites, were significantly down-regulated in strain Δ*laeA* when compared with the WT strain (Additional file 1: Table S13). Although secondary metabolites and their synthetic pathways need to be further explored in *M. thermophila*, these results demonstrate the important role of LaeA in the regulation of secondary metabolism in this fungus.

## Discussion

Filamentous fungi offer great potential advantages in the use of complex carbon sources and production of commodity chemicals at high concentration [2, 54]. Efficient cellulose degradation and utilization, and fungal growth are the prerequisites of cost-effective production of target products by fungal cell factory. In this study, we comprehensively investigated the mechanism of the putative methyltransferase LaeA regulating mycelium growth, sugar consumption, and cellulases expression, using *M. thermophila* as the test system. The deletion of *laeA* caused an obvious increase in sugar consumption and mycelium growth of *M. thermophila*. By combining functional genomic assays and molecular genetic analyses, we further identified four GRFs (*grf-1*, *grf-2*, *grf-3*, and *cre-1*) which help control mycelium growth connected with the LaeA regulatory network. In regard to the regulatory network of LaeA and connected GRFs, we can attribute the observed growth rate and glucose consumption phenotypes of strain Δ*laeA* and the four GRF mutants to their higher gluconeogenesis activities.

Utilization of gluconeogenic intermediates for the synthesis of structural polymers was preferential in eukaryotic cells [55–57]. Phosphoenolpyruvate carboxykinase (PCK) is the rate-limiting enzyme of gluconeogenesis and also a key regulator of TCA cycle flux [58]. In human melanoma and colon carcinoma, expression of PCK1 is markedly up-regulated to promote tumor growth [58]. In our study, *pck* expression in *M. thermophila* was notably increased owing to the deletion of *laeA*. The phenotypic analysis of mutants including overexpression of endogenous *pck* in the wild-type strain resulting in elevated mycelium growth in this fungus and deletion of *pck* in Δ*laeA* causing an obvious decrease in cell dry weight accumulation indicated that elevated gluconeogenesis activity partially contributed to the specific growth rate and glucose consumption of strain Δ*laeA*. According to recent reports, the expression of *pck* is precisely tuned by a regulatory network involving multiple layers, including epigenetic regulation, transcription regulation, and post-transcription regulation. In *A. nidulans*, transcriptional activators AcuK and AcuM function to activate the translation of the PCK-encoded gene *acuF* [59]. Herein, single mutation of the four GRF genes caused a reduction of expression level of *pck*, even though no significant binding between *pck* promoter and GRF proteins was observed, (Additional file 2: Fig. S7), suggesting that PCK is the core node of the metabolic network related to fungal vegetative growth, which is indirectly regulated by four GRFs.

Previous reports suggested that LaeA has been linked to changes in chromatin structure in *Apergillus* [22, 26, 60] and regulation of gene transcription through lysine or arginine methylation of histones [16, 17, 23–25]. In the present study, we found that deletion of *laeA* caused notably altered global methylation levels of H3K9me3 in *M. thermophila*, consistent with a ChIP-seq analysis in which 48.5% (1088 genes) of the 2242 genes bearing H3K9me3 showed decreased H3K9me3 levels in strain ∆*laeA*. However, H3K9me3 modification at the promoter regions of four GRF genes and *pck* did not correspond to their expression. Recent studies in *A. nidulans*, failed to identify a protein that is methylated by LaeA, but considerable automethylation was observed [30]. Dominguez et al. assumed that LaeA counteracts the trimethylation of H3K9 and the binding of heterochromatin protein to this repressive chromatin mark in *A. nidulans* [40]. In some fungi, *laeA* disruption reduced the H3K9 methylation levels of the key target genes [26]. On the other hand, LaeA function is dependent on methyltransferase domain in *M. thermophila*. These evidences suggests that LaeA and its orthologs may exert their function by binding to other regulator proteins rather than by directly methylating histones.

LaeA dynamically regulates the production of cellulases in *M. thermophila*. According to our transcriptomic analysis, the expression levels of CAZyme genes in Δ*laeA* were significantly lower in the early growth stage, but dramatically up-regulated thereafter when compared with those of WT strain. Cellulase-activated transcription factors, including *clr-1*, *clr-2*, *xlnR*, *col-26*, and *hac-1*, consistently followed similar trends at the transcriptional level, suggesting LaeA regulates cellulase production by influencing transcriptional levels of transcription factors. In *T. reesei*, Lae1, the LaeA ortholog, modulates cellulase gene expression, a process dependent on the function of the general cellulase regulator XYR1 [16, 20]. It was reported that in *P. oxalicum*, LaeA is necessary for the expression of cellulase activated by ClrB (an ortholog of Clr-2), where ClrB recruits PoTup1-Cyc8 complex and then PoTup1 recruits LaeA to regulate chromatin condensation of promoter regions for activating cellulolytic gene expression [21, 22]. However, an analysis of H3K4 and H3K9 methylation patterns in *lae1* mutants of *T. reesei* revealed that the LAE1-regulated expression of cellulase genes did not correlate with the changes in histone methylation and no enrichment of the histone marks was observed at the cellulolytic gene regulator Xyr1 gene locus [16]. Additionally, several hemicellulose encoding genes is positively regulated by ClrB but negatively regulated by LaeA in *P. oxalicum* [21, 22, 61]. These observations demonstrated LaeA might affect the recruitment of transcription-associated proteins by transcription factor to regulate the expression of gene encoding cellulolytic enzymes, which required further investigations to dissect the regulatory mechanism.

## Conclusion

In this study, we demonstrated that LaeA functions as a global regulator in *M. thermophila*, involving in sugar consumption and mycelium growth, cellulase expression, and secondary metabolites. LaeA negatively regulates sugar consumption and mycelium growth of *M. thermophila*. The analysis of transcriptomic profiling indicated four growth regulatory factors (Cre-1, Grf-1, Grf-2, and Grf-3) participated in the regulation of LaeA on fungal growth PCK is the core node of the metabolic network related to fungal vegetative growth, regulated by four GRFs and LaeA. Additionally, LaeA function is dependent on methyltransferase domain and involved in the process of H3K9 methylation. However, H3K9me3 modification at the promoter regions of four GRF genes and *pck* did not correspond to their expression. Also, LaeA dynamically regulates the expression of the genes encoding cellulolytic enzymes in *M. thermophila*, which was dependent on its methyltransferase activity.

## Materials and methods

### Strains and growth conditions

*M. thermophila* ATCC 42464 and its mutants were grown on Vogel’s minimal medium (VMM) supplemented with 2% (w/v) glucose at 35 °C to obtain mature conidia. Antibiotic was added when needed to screen for transformants. *Escherichia coli* Mach-T1 were used for vector manipulation and propagation. Strains were cultivated in Luria–Bertani (LB) medium with 100 µg/mL ampicillin or 50 µg/mL kanamycin for plasmid selection.

### Construction of *M. thermophila* mutants

To delete target gene in *M. thermophila*, the gene coding region was replaced by selecting marker gene expression cassette. This process needs three DNA fragments: Cas9, sgRNA, and Donor. Cas9-expressing cassette was amplified from plasmid p0380-bar-P*tef1*-Cas9-TtprC [10, 11] using the primer pairs (Additional file 1: Table S1). The target sites of genes were designed by sgRNACas9 tool [11]. The sgRNA was constructed by connecting U6 promoter, genes target sites and gRNA by fusion-PCR. About 1 kb of the up- and downstream non-coding region of target genes were amplified from genomic DNA of *M. thermophila*. The two resultant PCR fragments as well as the P*trpc*-*neo* or P*trpc*-*bar* fragment were connected to generate donor sequence by Gibson Assembly and then ligated into an XbaI/EcoRV restricted vector pPk2-Neo-GFP. The donor DNA included pPk2-*laeA*-*neo*, pPk2-*grf1*-*bar*, pPk2-*grf2*-*bar*, pPk2-*grf3*-*bar*, pPk2-*cre1*-*bar*, and pPk2-*pck*-*bar*, and corresponding sgRNAs were U6-*laeA*-sgRNA, U6-*grf1*-sgRNA, U6-*grf2*-sgRNA, U6-*grf3*-sgRNA, U6-*cre1*-sgRNA, and U6-*pck*-sgRNA. To complement Δ*laeA* with LaeAM1, LaeAM2, or LaeAM3, corresponding donors DNA sequence and sgRNA targeting *neo* used to replace *laeA* were constructed as mentioned above.

For the construction of plasmids overexpressing target genes, *laeA* (Mycth_2294559), *pck* (Mycth_2315623), and *fbp* (Mycth_2306943) were amplified using *M. thermophila* cDNA as the template and under control of the strong constitutive promoter P*tef1*.

### Fungal transformation

The PEG-mediated transformation of *M. thermophila* protoplasts was performed. For gene overexpression, 10 µg linearized plasmid was transformed into *M. thermophila* protoplasts and transformants were selected on plates containing antibiotics. For gene replacement, sgRNA and donor DNA of target genes were mixed with the Cas9-expression PCR cassette and co-transformed into *M. thermophila*. The putative transformants were selected with antibiotics and confirmed via PCR amplification of the transgene with paired primers (Additional file 1: Table S1).

### Confocal fluorescence microscopy

To localize GFP-fused proteins using microscopy, strains were inoculated into 1 × VMM with 2% glucose and grown for 16 h at 45 °C. Microscopic observations were performed using an Olympus BX51 fluorescence microscopy system and images were processed using ImageJ software.

### Growth characters and biochemical assays

To assay the growth phenotypes of strain WT and Δ*laeA*, we inoculated 10^5^ spores into agar plate of 1 × VMM supplemented with 2% (w/v) carbon resources and take pictures after 5 d of the cultivation at 35 °C.

To evaluate sugar consumption and biomass accumulation, mature spores were inoculated into 50-mL 1 × VMM supplemented with 2% glucose in a 250-mL Erlenmeyer flask and incubated at 45 °C and 150 rpm on a rotary shaker. Samples were taken at different intervals. Glucose concentration was measured using HPLC with an e2695 instrument (Waters, Manchester, UK) and cell dry weigh was detected after dried to constant weight.

The protein concentration in the supernatant was determined using the Bio-Rad protein assay kit (Bio-Rad, Hercules, CA, USA) with bovine serum albumin used as the standard. The absorbance of the mixture was measured at 595 nm. Azo-CM-cellulose assay kit (Megazyme) was used to determine endo-glucanase (CMCase) activity.

### Analysis of transcriptomic data

*M. thermophila* wild-type strain and its mutant were cultured in 1 × VMM with 2% glucose (Sampled at 24 h) or Avicel (Sampled at 2 d, 4 d, or 6 d) as the carbon source. Mycelia were collected by filtration, immediately homogenized in liquid nitrogen and stored at − 80 °C until used for RNA extraction. Total RNA was extracted with a modification of the method described previously, using TRIzol reagent [2, 62]. Genomic DNA contamination was eliminated by an additional clean-up using the RNeasy mini kit (Qiagen), according to the manufacturer’s RNA clear-up instructions. RNA integrity and concentration were determined using Nanodrop and agarose gel electrophoresis.

Complementary DNA libraries were prepared with standard protocols from Illumina and sequenced on an Illumina platform. The clean reads were mapped against *M. thermophila* ATCC42446 *genome* [7] using Tophat (v2.0.8b) [63]. The counts of reads mapped to unique exons were calculated by Htseq-count (V0.13.5) with SAM files and genome annotation file as input and used for normalizing transcript abundance (RPKM, reads per kilobase per million mapped reads). Different gene expression analysis was carried out using the DESeq package [64]. Inter-sample correlation analyses were conducted for strain WT and Δ*laeA*. Unless otherwise noted, genes with |log2 FoldChange|≥ 1, RPKM ≥ 20 (at least one sample), and DESeq *P*-adj value < 0.05, were considered significantly differentially expressed between two samples.

### Assays of electrophoretic mobility shift

Fragments encoding the DNA-binding domains of four transcription factors were amplified using *M. thermophila* cDNA as template. Then resulting fragments were inserted into a BamHI/XhoI restricted vector pGEX-4T-1 (GE Healthcare) to form pGEX-GRFs. The plasmids were subsequently introduced into *E. coli* BL21 (DE3) for protein expression. *E. coli* BL21 (DE3) harboring pGEX-GRFs were grown at 37 °C in 100 mL LB medium to an OD600 of 0.4. Isopropyl *β*-d-1-thiogalactopyranoside (IPTG) was then added with a final concentration of 0.5 mM, and the cultures were incubated for an additional 20 h at 15 °C. The cells were harvested by centrifugation and re-suspended in phosphate-buffered saline followed by sonication, after which the insoluble material was removed by centrifugation at 8000*g* for 10 min. The glutathione S-transferase (GST)-fused protein was purified using a BeaverBeads™ GSH (Beaver, China) according to the manufacturer’s manual.

The probes fragments were designed to cover the promoter regions of the corresponding genes. The positions relative to the translation start site were shown in Additional file 1: Table S1. The PCR product was purified and the concentration was determined and diluted to 10 ng/μL. The subsequent binding experiments were performed using a modified gel mobility shift assay. In each EMSA, different recombinant proteins were incubated with a constant amount (10 ng) of the DNA probes individually at 25 °C for 30 min.

### Assays of global histone H3 methylation levels

The global histone H3K4/H3K9/H3K27 methylation are measured by EpiQuik™ Global Histone H3K4/H3K9/H3K27 Methylation Assay Kit (P-3017/P-3018/P-3020, Epigentek). Briefly, histone protein from strains WT and Δ*laeA* was stably spotted on the strip wells, and the methylated histone H3K4/H3K9/H3K27 can be recognized with the corresponding high-affinity antibody. The amount of H3K4/H3K9/H3K27 methylation (me1, me2, and me3) levels could be quantified through horseradish peroxidase (HRP)-conjugated secondary antibody-color development system and was measured at 450 nm.

### Histone extraction and Western blotting analysis

Histone extraction was performed by the modified protocol from fungal histone extraction assay (Biolabs, Beijing, China). Briefly, mycelia were ground in liquid nitrogen using a mortar and pestle, dissolved in PBS buffer containing 1 mM PMSF. Then supernatant was collected after low-speed centrifugation. Cell nuclei as well as other small organelles were in the supernatant. The nuclei were then precipitated by high-speed centrifugation, resuspended in 0.4 M hydrochloric acid. Use a sonicator to disrupt the nuclei, following by adding acetone to precipitate proteins overnight in 4℃. The precipitates obtained by centrifugation were histones.

The histone of *M. thermophila* strains grown on 2% glucose for 24 h was extracted for western blotting analysis using H3 antibody (OM256785, OmnimAbs), H3K4me3 antibody (ACTIVE MOTIF, 39,159), and H3K9me3 antibody (ab8898, abcam) as the primary antibodies, respectively, and the anti-rabbit IgG HRP antibody (M21002, Abmart) as the secondary antibodies.

### Chromatin immunoprecipitation analysis

Chromatin Immunoprecipitation (ChIP) was performed by the modified protocol from Simple ChIP Plus Enzymatic Chromatin IP Kit (Magnetic Beads, #9005). Briefly, the mycelia were fixed with 1% formaldehyde for 15 min at 45 °C with shaking and then stopped with glycine at a final concentration of 125 mM. Cross-linked tissues were ground and resuspended at 1 g/mL in lysis buffer containing 1 mM PMSF, 1 μg/ml pepstatin A and 1 μg/mL leupeptin. Chromatin was sheared by sonication to 100–1000-bp fragments. The chromatin immunoprecipitation was performed using 4 μg H3K9me3 antibody (ab8898, abcam) per 2 mg protein. After DNA extraction, the pellets were resuspended in 300 μL of DNase-free water.

For ChIP-seq, library construction was performed by E-GENE Corporation (Shenzhen, China), and pair-end sequencing was performed on Illumina NovaSeq6000 platform. After pass quality control with Trim Galore (version 3.2), paired-reads were mapped to *M. thermophila* genome using Bowtie2 (version 2.3.5.1). Only unique mappers were retained for downstream analyses. MACS2 (version 2.2.7.1) was employed for peak calling Mapped read density was calculated over 10 bp bins and normalized by RPGC using bamCoverage from deepTools (version3.5.1), denoised with sliding window (window size is 100 nt and step is 10 nt) and visualized with the Integrative Genomics Viewer (IGV).

### Statistical significance tests

Unless otherwise noted, statistical significance was tested using a one-tailed homoscedastic (equal variance) *t* test. All *p *values were generated using Microsoft Excel 2013 (Microsoft Corporation). n.s. indicates no statistical significance; * represents a *p* value < 0.05; **represents a *p* value < 0.01; and *** represents a *p* value < 0.001.

## Supplementary Information

**Additional file 1: Table S1.** Primers used in this study. **Table S2.** The profiles of RNA-seq reads mapped to the genome of *M. thermophila* and the differential expression analysis. **Table S3.** Genes showing significantly differential transcriptional levels in strain *∆laeA* on glucose compared to the wild-type strain. **Table S4.** KEGG analysis of the 337 genes with up-regulated expression levels in strain *∆laeA* on glucose. **Table S5.** Transcriptional profile of genes involved in metabolic pathway in *M. thermophila*. **Table S6.** Transcription factors showing significantly differential transcriptional levels in strain *∆laeA* on glucose as compared to the wild-type strain. **Table S7.** Genes showing significantly differential transcriptional levels in strains Δ*cre-1*, Δ*grf-1*, Δ*grf-2*, and Δ*grf-3* on glucose compared to the wild-type strain. **Table S8.** KEGG analysis of genes showing significantly up-regulated transcriptional levels in all strains (∆*cre-1*, Δ*grf-1*, Δ*grf-2*, and Δ*grf-3*), compared to the wild type. **Table S9.** The analysis of H3K9 methylation level in Δ*laeA* compared to WT by ChIP-seq. **Table S10.** Genes showing significantly differential transcriptional levels in strain Δ*laeA* on Avicel for 2 d compared to the WT strain and functional category analysis of the genes expressed more highly in strain Δ*laeA* (p values < 0.005). **Table S11.** Genes showing significantly differential transcriptional levels in strain Δ*laeA* on Avicel for 4 d compared to the WT strain and functional category analysis of the genes expressed more highly in strain Δ*laeA* (p values < 0.05). **Table S12.** Transcriptional levels of CAZyme genes in strain Δ*laeA* on Avicel for 2d and 4 d. **Table S13.** Transcriptomic profiles of the genes related to the secondary metabolism in the strain Δ*laeA* when response to Avicel.

**Additional file 2: Fig. S1.** Overview of sequence analysis of LaeA. **a** Maximum-likelihood trees of LaeA homologs in fungi. The tree had a log likelihood of -14608. The aLRT support provides an estimate of branch reliability and can be interpreted as bootstrap percentages. **b** Sequence alignment of *M. thermophila* LaeA and its orthologs in other fungi. Bars are used to denote the amino acids comprising the four common methyltransferase motifs of seven-β-strand methyltransferases. **Fig. S2.** Time course of glucose consumption of *M. thermophila* strains WT and Δ*laeA* grown on 20 g/L glucose. **Fig. S3.** Assay of spore germination and mycelium growth. Morphology of strain Δ*laeA* in comparison to the WT strain. All strains were cultivated on 1 × VMM plus 2% glucose at 45 °C. Fungal spores and hyphae were observed under microscopy. **Fig. S4.** Sugar consumption of strains OE_*pck* overexpressing *pck* and OE_*fbp* overexpressing *fbp* when growth on 20 g/L glucose. **Fig. S5.** Phenotypic analysis of the strain ∆*laeA*∆*laeB* on Avicel. **a** Expression level of all LaeA-like methyltransferase genes in Δ*laeA* strain in Vogel’s MM with 2% Avicel. **b** Total extracellular protein concentration and endo-glucanase activity of strain Δ*laeA*Δ*laeB* in Vogel’s MM with 2% Avicel. **Fig. S6.** Affect of *laeA* deletion on secondary metabolism in *M. thermophila*. **a** Phenotype of strains WT and Δ*laeA* grown on Avicel. **b** Heatmap analysis of polyketide synthase (PKS) and nonribosomal peptide synthetase (NRPS) genes with differentially expressed levels in strain Δ*laeA*, compared to strain WT when growth on Avicel. **Fig. S7.** Electrophoretic mobility shift assays of binding of Cre-1, Grf-1, Grf-2, and Grf-3 to upstream DNA regions of gluconeogenesis key enzyme-encoding gene *pck*.

## Data Availability

The data supporting the findings of this work are available within the paper and the supplementary materials. The data of RNA-seq and ChIP-seq have been uploaded in Gene Expression Omnibus (GEO, accession number: GSE213576) at the National Center for Biotechnology Information (NCBI). The data sets generated and analyzed during this study are available from the corresponding author upon request.
