# Supplementary material for: The putative methyltransferase LaeA regulates mycelium growth and cellulase production in Myceliophthora thermophila
Source: Biotechnol Biofuels Bioprod. 2023 Apr 3;16:58. doi: 10.1186/s13068-023-02313-3 (PMC10071736; doi:10.1186/s13068-023-02313-3)
Supplement: Supplementary file 2 — Additional file 2: Fig. S1. Overview of sequence analysis of LaeA. a Maximum-likelihood trees of LaeA homologs in fungi. The tree had a log likelihood of -14608. The aLRT support provides an estimate of branch reliability and can be interpreted as bootstrap percentages. b Sequence alignment of M. thermophila LaeA and its orthologs in other fungi. Bars are used to denote the amino acids comprising the four common methyltransferase motifs of seven-β-strand methyltransferases. Fig. S2. Time course of glucose consumption of M. thermophila strains WT and ΔlaeA grown on 20 g/L glucose. Fig. S3. Assay of spore germination and mycelium growth. Morphology of strain ΔlaeA in comparison to the WT strain. All strains were cultivated on 1 × VMM plus 2% glucose at 45 °C. Fungal spores and hyphae were observed under microscopy. Fig. S4. Sugar consumption of strains OE_pck overexpressing pck and OE_fbp overexpressing fbp when growth on 20 g/L glucose. Fig. S5. Phenotypic analysis of the strain ∆laeA∆laeB on Avicel. a Expression level of all LaeA-like methyltransferase genes in ΔlaeA strain in Vogel’s MM with 2% Avicel. b Total extracellular protein concentration and endo-glucanase activity of strain ΔlaeAΔlaeB in Vogel’s MM with 2% Avicel. Fig. S6. Affect of laeA deletion on secondary metabolism in M. thermophila. a Phenotype of strains WT and ΔlaeA grown on Avicel. b Heatmap analysis of polyketide synthase (PKS) and nonribosomal peptide synthetase (NRPS) genes with differentially expressed levels in strain ΔlaeA, compared to strain WT when growth on Avicel. Fig. S7. Electrophoretic mobility shift assays of binding of Cre-1, Grf-1, Grf-2, and Grf-3 to upstream DNA regions of gluconeogenesis key enzyme-encoding gene pck. [file 13068_2023_2313_MOESM2_ESM.pdf]

The putative methyltransferase LaeA regulates mycelium growth and cellulase production in *Myceliophthora thermophila*

Zhao *et al.*

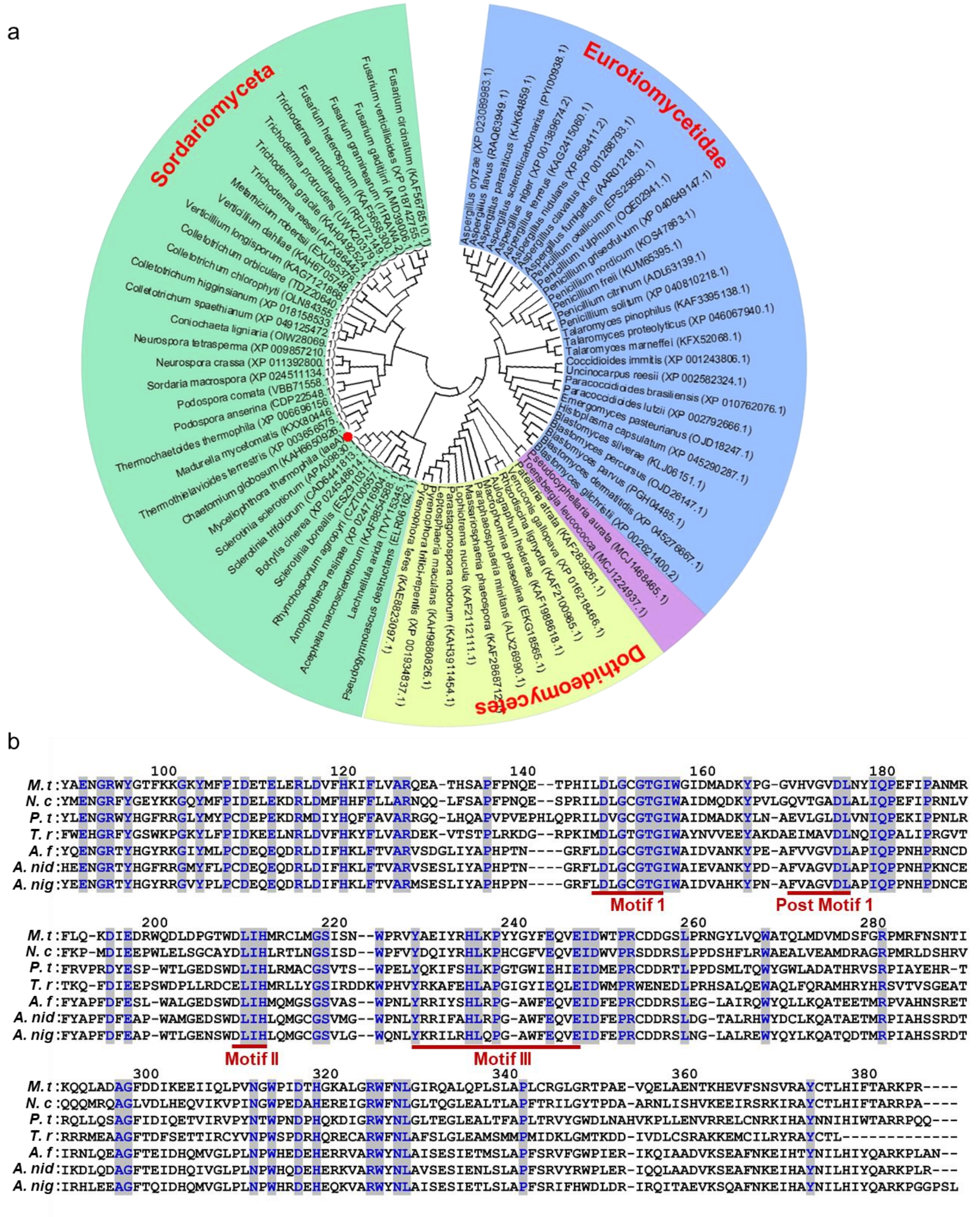

Fig. S1 Overview of sequence analysis of LaeA. **a** Maximum-likelihood trees of LaeA homologs in fungi. The tree had a log likelihood of -14608. The aLRT support provides an estimate of branch reliability and can be interpreted as bootstrap percentages. **b** Sequence alignment of *M. thermophila* LaeA and its orthologs in other fungi. Bars are used to denote the amino acids comprising the four common methyltransferase motifs of seven-β-strand methyltransferases.

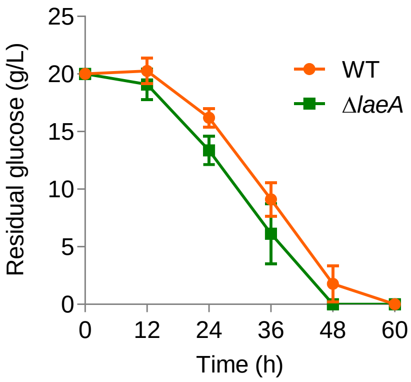

Fig. S2 Time course of glucose consumption of *M. thermophila* strains WT and Δ*laeA* grown on 20 g/L glucose.

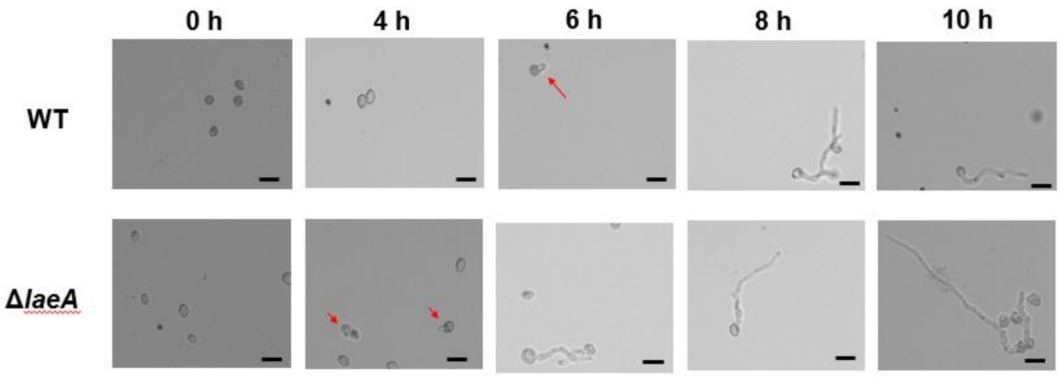

Fig. S3 Assay of spore germination and mycelium growth. Morphology of strain Δ*laeA* in comparison to the WT strain. All strains were cultivated on 1×VMM plus 2% glucose at 45°C. Fungal spores and hyphae were observed under microscopy.

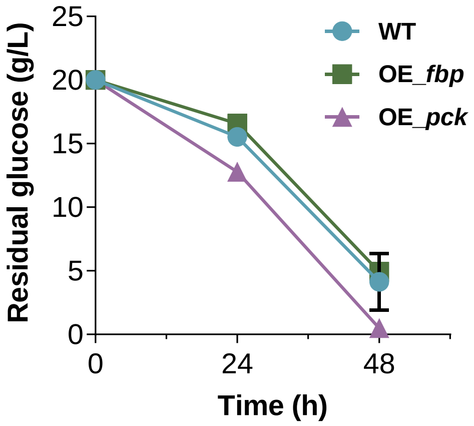

Fig. S4 Sugar consumption of strains OE_*pck* overexpressing *pck* and OE_*fbp* overexpressing *fbp* when growth on 20 g/L glucose.

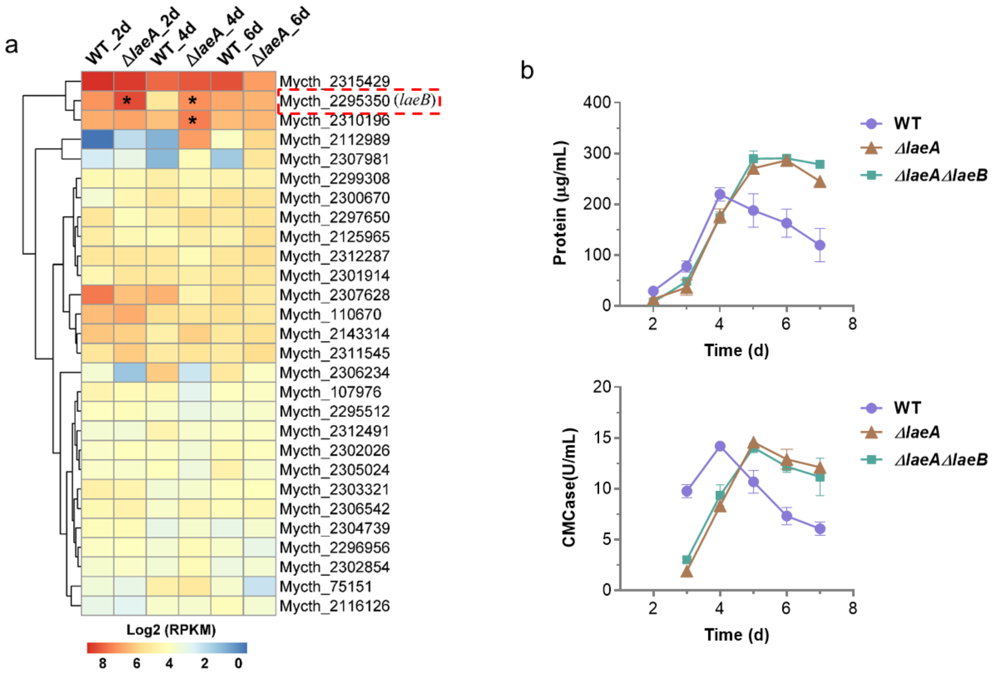

Fig. S5 Phenotypic analysis of the strain Δ*laeA*Δ*laeB* on Avicel. **a** Expression level of all LaeA-like methyltransferase genes in Δ*laeA* strain in Vogel's MM with 2% Avicel. **b** Total extracellular protein concentration and endo-glucanase activity of strain Δ*laeA*Δ*laeB* in Vogel's MM with 2% Avicel.

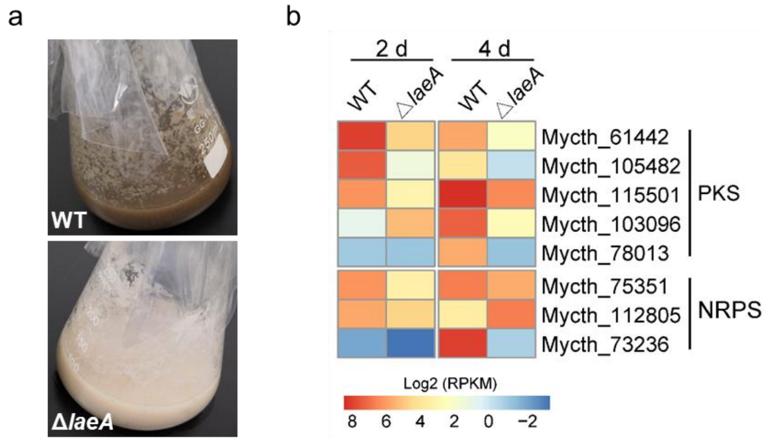

Fig. S6 Affect of *laeA* deletion on secondary metabolism in *M. thermophila*. **a** Phenotype of strains WT and Δ*laeA* grown on Avicel. **b** Heatmap analysis of polyketide synthase (PKS) and nonribosomal peptide synthetase (NRPS) genes with differentially expressed levels in strain Δ*laeA*, compared to strain WT when growth on Avicel.

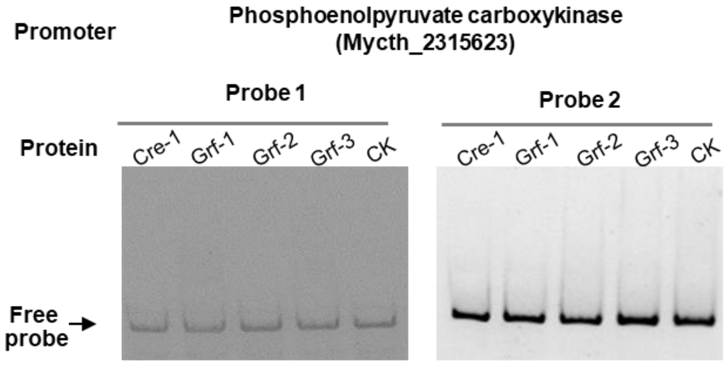

Fig. S7 Electrophoretic mobility shift assays of binding of Cre-1, Grf-1, Grf-2, and Grf-3 to upstream DNA regions of gluconeogenesis key enzyme-encoding gene *pck*.
